# Dietary Bile Acids Enhance Growth, and Alleviate Hepatic Fibrosis Induced by a High Starch Diet via AKT/FOXO1 and cAMP/AMPK/SREBP1 Pathway in *Micropterus salmoides*

**DOI:** 10.3389/fphys.2019.01430

**Published:** 2019-11-19

**Authors:** Huanhuan Yu, Lulu Zhang, Pei Chen, Xiaofang Liang, Aizhi Cao, Juan Han, Xiufeng Wu, Yinhua Zheng, Yuchang Qin, Min Xue

**Affiliations:** ^1^National Aquafeed Safety Assessment Center, Feed Research Institute, Chinese Academy of Agricultural Sciences, Beijing, China; ^2^Institute of Animal Sciences, Chinese Academy of Agricultural Sciences, Beijing, China; ^3^Institute of Food and Nutrition Development, Ministry of Agriculture and Rural Affairs, Beijing, China; ^4^Key Laboratory of Feed Biotechnology, Ministry of Agriculture and Rural Affairs, Feed Research Institute, Chinese Academy of Agricultural Sciences, Beijing, China

**Keywords:** bile acids, inflammation, apoptosis, liver health, glucose and lipid metabolism, *Micropterus salmoides*

## Abstract

A 10-week feeding trial was conducted to investigate the effects of dietary bile acids (BA) on growth, glucose and lipid metabolism, liver histopathology, and the underlying regulation mechanism on AKT/FOXO1 (forkhead box O1) and cAMP/AMPK/SREBP1 (sterol regulatory element-binding protein 1) pathway in largemouth bass (*Micropterus salmoides*) fed with a high starch diet. Six experimental diets were prepared with BA levels at 0 (B0), 80 (B80), 160 (B160), 240 (B240), 300 (B300), and 600 (B600) mg/kg in a basal diet with 18.7% starch. Each diet was fed to six replicates with 30 fish (6.17 ± 0.03 g) in each tank. The highest weight gain rate (WGR) was observed in B300 group and the optimal level of BA was estimated at 475 mg/kg by a monistic cubic equation regression analysis. Dietary BA inclusion decreased hepatosomatic index (HSI) and hepatic lipid content significantly. The fish in B300 group clearly showed alleviated hepatic fibrosis, but more steatohepatitis symptoms diagnosed with various histopathological and immunofluorescence analysis. 10 out of 12 samples were observed hepatic fibrosis in B0 group while only two fibrosis samples in B300 group. The promoted liver histopathology by dietary BA was related to improved glucose and lipid metabolism. Dietary BA inhibited the expression of G6Pase by activating AKT and reducing FOXO1 transcription, which improved the regulation ability of gluconeogenesis, activated cAMP/AMPK and repressed SREBP1 transcription to inhibit hepatic lipogenesis, which prevented hepatic lipid accumulation. In conclusion, dietary BA enhanced the growth and alleviated liver fibrosis induced by a high starch diet to steatohepatitis/recovery symptom via improving glucose and lipid metabolism, which regulated by AKT/FOXO1 and cAMP/AMPK/SREBP1 pathway in largemouth bass.

## Introduction

Fish, particularly the carnivorous species have limited capability of using carbohydrate as energy sources (Hemre et al., 2002; Gong et al., 2015). The poor adaptation to high dietary carbohydrate loads in carnivorous fish may induce glycogen accumulation in liver tissue and further initiate glucose and lipid metabolic disorder in fish (Amoah et al., 2008; Yu et al., 2018). The largemouth bass (*Micropterus salmoides*) is a carnivorous species which has become one of the most commercially important species in China. High dietary digestible carbohydrate induced prevalent metabolic liver disease (MLD) in largemouth bass. Previous studies suggested that the starch (the main source of digestible carbohydrate in aquafeeds) inclusion level in the diet should be lower than 10% to ensure the liver health of largemouth bass (Xu et al., 2016; Ma et al., 2019). However, traditional floating fish feeds generally require a minimum of 20% starch to create sufficient expansion and low densities for high buoyancy properties. Therefore, the low-starch inclusion has made feed processing and product durability a challenge (Riaz and Rokey, 2012). In addition, low-starch inclusion leads to increased feed costs and processing energy consumption.

It was well-established that bile acids (BA) played vital roles in cholesterol elimination, stimulation of bile flow and biliary secretion, feedback inhibition of BA and cholesterol synthesis and promotion of intestinal digestion and absorption of dietary lipids, and fat-soluble nutrients (Olsen et al., 2005; de Aguiar Vallim et al., 2013). BA have different molecular forms. In human, both cholic acid (CA) and chenodeoxycholic acid (CDCA) are primary BA synthesized in hepatocytes while deoxycholic acid (DCA), lithocholic acid (LCA), ursocholic acid (UCA), and ursodeoxycholic acid (UDCA) are secondary BA converted by intestinal microbiota (Dawson and Karpen, 2015). Among them, CA, CDCA, DCA, and UDCA have been approved as therapeutic agents for the hepatobiliary disease (Ashby et al., 2018). Besides, BA were prospective candidates for therapy in steatohepatitis given the regulation in their own homeostasis as well as cholesterol and lipid homeostasis (Nie et al., 2012; Li and Chiang, 2014; Trauner et al., 2017). In 2016, BA was approved as a new feed additive in China. However, the studies of BA in fish were limited and most data focused on growth performance, body composition, nutrients utilization, and antioxidant status (Alam et al., 2001; Yamamoto et al., 2007; Sun et al., 2014; Du et al., 2017). The regulation mechanisms of exogenous BA on nutrients metabolism and MLD in fish are not clear yet.

AKT (AKT serine/threonine kinase) is one of the most important and multi-functional kinases, regulating various cellular functions like survival, metabolism, and proliferation, etc. (Manning and Toker, 2017). AKT/FOXO1 (forkhead box O1) was demonstrated to play an important part in improving glucose homeostasis by regulating gluconeogenesis (Gayen et al., 2009; Lin and Accili, 2011). AMPK (adenosine 5′-monophosphate (AMP)-activated protein kinase), an important energy sensor, has a vital function to maintain cellular energy homeostasis (Hardie et al., 2012). It was reported that cAMP (cyclic adenosine monophosphate)/AMPK/SREBP (sterol regulatory element-binding protein) pathway plays a very important regulatory role in metabolic diseases (fatty liver, obesity, diabetes, etc.) (Lee et al., 2014, 2015; Li et al., 2018), in which, AMPK/SREBP pathway was widely studied and recognized to play a key role in the development of MLD related to energy metabolism disorder (Li et al., 2011; Lee et al., 2015; Dihingia et al., 2018). Until now, there were no reports of BA functions on nutrient metabolism and related disease by regulating the AKT and AMPK pathway in fish.

The objectives of the present study were to investigate effects of dietary BA on growth performance, glucose and lipid metabolism and liver histopathology based on an 18.7% of starch inclusion (meeting requirement for the floating feed processing) diet in largemouth bass and the underlying regulating mechanism on AKT/FOXO1 and cAMP/AMPK/SREBP1 (sterol regulatory element-binding protein 1) signal pathway.

## Materials and Methods

### Experimental Diets

Six isonitrogenous (46.6%) and isoenergetic (20.1 MJ/kg) experimental diets with 18.7% starch were prepared with BA (supplied by Shandong Longchang Animal Health Care Co., Ltd., Dezhou, China, with 8.0% hyocholic acid, 70.9% hyodeoxycholic acid, and 20.2% CDCA) levels at 0 (B0), 80 (B80), 160 (B160), 240 (B240), 300 (B300), and 600 (B600) mg/kg, respectively. Each diet was processed into 2 mm diameter floating pellets under the following extrusion condition: feeding section (90°C/5 s), compression section (130°C/5 s), and metering section (150°C/4 s) using a Twin-screwed extruder (EXT50A, YANGGONG MACHINE, China). All diets were air-dried at room temperature (23–30°C) and stored at −20°C until use. The diet formulation and analyzed biochemical compositions were shown in Table 1.

**TABLE 1:** Formulation and compositions of basal diets (air-dry basis, %).

**Ingredients**	**Basal diet**
Fish meal	18.0
Soybean protein concentrate	19.0
Soybean meal	10.0
Krill meal	4.00
Wheat gluten	14.0
Wheat flour	20.23
Yeast extract	1.00
Soy lecithin (60%)	2.00
Fish oil	8.00
Ca(H_2_PO_4_)_2_	2.17
*DL*-Met (98%)	0.20
Vitamin and mineral premix	1.40
Total	100
**Analyzed chemical compositions (dry matter basis, %)**	
Crude protein	50.8
Crude lipid	13.6
Starch	18.7
Ash	8.40
Gross energy/(MJ/kg)	20.1

*Fish meal: Triple Nine Fish Protein Co., Ltd. (Denmark); Soybean protein concentrate: Yihai Kerry Investment Co., Ltd. (China); Soybean meal: Yihai Kerry Investment Co., Ltd. (China); and Krill meal: Liaoning Pelagic Fishery Co., Ltd. (China). The vitamin and mineral premix are provided as following (mg/kg diets): VA 20, VB_1_ 10, VB_2_ 15, VB_6_ 15, VB_12_ 8, VE 400, VK_3_ 20, VD_3_ 10, niacinamide 100, vitamin C phosphate (35%) 1000, inositol 200, calcium pantothenate 40, biotin 2, folic acid 10, choline chloride (50%) 4000, corn gluten meal 150 mg; CuSO_4_⋅5H_2_O 10, FeSO_4_⋅H_2_O 300, ZnSO_4_⋅H_2_O 200, MnSO_4_⋅H_2_O 100, KIO_3_ (10%) 80, Na_2_SeO_3_ (10%) 10, NaCl 100, CoCl_2_⋅6H_2_O (10%) 5, MgSO_4_ 2 000, powdered zeolite 4995, and 200 Tertiary Butylhydroquinone (TBHQ). TBHQ was pre-added into fish oil as antioxidant. Bile acids: supplied by Shandong Longchang Animal Health Care Co., Ltd. (Dezhou, China), with 8.0% hyocholic acid, 70.9% hyodeoxycholic acid, and 20.2% chenodeoxycholic acid. Bile acids were added and well mixed in premix at levels of 0, 80, 160, 240, 300, and 600 mg/kg, respectively.*

### Experimental Fish, Feeding, and Sampling

The largemouth bass was obtained from the Sanshui platinum Aquafarm (Foshan, Guangdong, China). All fish were acclimated and fed the control experimental diet (B0) for 2 weeks before the trial. Fish (6.17 ± 0.03 g) were selected and distributed into 36 tanks (256 L) after 24 h starvation with 30 fish per tank and six tanks per treatment. Fish were fed to apparent satiation twice daily at 08:00 and 16:00 for 70 days. The water temperature was maintained at 22–24°C, pH = 7–8, dissolved oxygen (DO) > 6.0 mg/L and NH_4_-N < 0.5 mg/L. Aeration was supplied to each tank 24 h per day. The photoperiod was 12D: 12L and the light intensity was 400 lx. During the feeding period, recording the feed consumption.

At the end of the feeding trial, fish fasted for 24 h. Then, the fish in each tank were counted and batch weighed to calculate survival rate (SR), final mean body weight (FBW), weight gain rate (WGR), specific growth rate (SGR), voluntary feed intake (VFI), and feed conversion ratio (FCR). All the sampled fish were anesthetized with chlorobutanol (300 mg/L, SINOPHARM, China) before sampling. Individual body weight, viscera, and liver weight of three fish in each tank were recorded to calculate viscerosomatic index (VSI) and hepatosomatic index (HSI). Blood samples were drawn from the caudal part of the sedated fish using anticoagulant syringes with 2% sodium fluoride (SINOPHARM, China) and 4% potassium oxalate (SINOPHARM, China). Blood samples were centrifuged at 1500 g for 10 min at 4°C to obtain plasma, and then stored at −80°C for analysis of hematological parameters. Two liver samples near to the bile duct in each tank were put into 1.5 mL RNase-free tubes (Axygen, United States), fast frozen in liquid nitrogen and then stored at −80°C for mRNA level and western blot analysis, or fixed in 4% paraformaldehyde no longer than 48 h for histology determination. Two liver samples in each tank were put into 1.5 mL tubes, fast frozen in liquid nitrogen and stored at −80°C for detection of hepatic cAMP. Livers from another ten fish in each tank were pooled into ziplock bags and then stored at −20°C for the assay of moisture and crude lipid. Whole bodies of four fish per tank were collected into plastic bags and then stored at −20°C for the assay of moisture, crude protein and crude lipid to calculate productive lipid value (PLV) and productive protein value (PPV).

### Biochemical Analysis

All biochemical analyses were carried out according to the standard procedure (Association of Official Analytical Chemists [AOAC], 2006). The dry matter was analyzed by drying the samples to a constant weight at 105°C. Crude protein was determined using a KjeltecTM 2300 Unit (Foss, Denmark) by the method of Kjeldahl, and the crude protein content was estimated by multiplying nitrogen by 6.25. Crude lipid was analyzed by acid hydrolysis with a Soxtec System HT 1047 Hydrolyzing Unit (Foss, Denmark), followed by Soxhlet extraction using a Soxtec System 1043 (Foss, Denmark). Starch was analyzed by colorimetric using 3,5-dinitrosalicylic acid at a wavelength of 540 nm (TU-1900, PERSEE, China). Ash was analyzed by combustion in a muffle furnace (CWF1100, Carbolite, United Kingdom) at 550°C for 16 h. Gross energy was determined using an IKAC2000 Calorimeter (C2000, IKA, Germany).

### Hematological Parameters and Hepatic cAMP Content

Plasma alanine aminotransferase (ALT), aspartate aminotransferase (AST), total protein (TP), albumin (ALB), urea nitrogen (BUN), glucose (GLU), triglyceride (TG), total cholesterol (TC), and high-density lipoprotein cholesterol (HDL-C) were determined by assay kits following the protocols given by the supplier (Nanjing Jiancheng Bioengineering Institute, China) (Yu et al., 2014). Hepatic cAMP content was detected by enzyme-linked immunosorbent assay (Elabscience Biotechnology Co., Ltd., China) (You et al., 2019).

### RNA Isolation, Reverse Transcription, and Real-Time Quantitative PCR (RT-qPCR)

Total RNA was isolated from liver using RNAiso Plus reagent (Takara, Japan). Then, the extracted RNA was spectrophotometrically quantified using a NanoDrop 2000 (Thermo Fisher Scientific, United States). The integrity of the RNA was detected by electrophoresing on a 1% denaturing agarose gel. For each reverse transcription reaction, 1.0 μg of total RNA was first treated with gDNA Eraser to remove genomic DNA contaminants and then subjected to cDNA synthesis by reverse transcription in a 20 μL volume using the PrimeScript RT reagent Kit (Takara, Japan).

The core fragments of all the genes were obtained from the database of RNA-Seq. The sequencing data had been submitted to the NCBI Sequence Read Archive (SRA) under the accession numbers SRR10158532 and SRR10158533. EF1α (elongation factor 1α, GenBank accession no. KT827794), a housekeeping gene whose expression was found to be unaffected by the treatment in the present experiment, was used as an endogenous reference to normalize the template amount. The gene-specific primers used for mRNA quantification by RT-qPCR were shown in Table 2. Serial dilutions of cDNA generated from liver tissues were used to make a standard curve to determine the amplification efficiency (*E*-values) of reference and target genes. The *E*-values ranged from 90.9 to 113.6% (Table 2). The RT-qPCR analysis was performed using a CFX96TM Real-Time System (Bio-Rad, United States) in a 20 μL reaction volume containing iTaqTM Universal SYBR^®^ Green Supermix (Bio-Rad, United States).

**TABLE 2:** Primer sequences for RT-qPCR.

**Gene**	**Primers**	**Sequence 5′-3′**	**Product size (bp)**	***E*-values (%)**	**Tm (°C)**
EF1α	F	TGCTGCTGGTGTTGGTGAGTT	147	102.8	60.4
	R	TTCTGGCTGTAAGGGGGCTC			
TNFα	F	CTTCGTCTACAGCCAGGCATCG	161	105.7	63.0
	R	TTTGGCACACCGACCTCACC			
IL1β	F	CGTGACTGACAGCAAAAAGAGG	166	101.3	59.4
	R	GATGCCCAGAGCCACAGTTC			
IL8	F	CGTTGAACAGACTGGGAGAGATG	112	111.8	64.9
	R	AGTGGGATGGCTTCATTATCTTGT			
IL10	F	CGGCACAGAAATCCCAGAGC	119	113.6	62.1
	R	CAGCAGGCTCACAAAATAAACATCT			
IL11β	F	TTCCCAACAGACAGATGAAGAACTC	182	113.3	60.0
	R	TGCCTGTGTTCAGCCAGTCAA			
TGFβ1	F	GCTCAAAGAGAGCGAGGATG	118	95.6	59.0
	R	TCCTCTACCATTCGCAATCC			
CASP8	F	GAGACAGACAGCAGACAACCA	119	103.1	62.1
	R	TTCCATTTCAGCAAACACATC			
CASP10	F	CAAACCACTCACAGCGTCTACAT	146	100.2	56.0
	R	TGGTTGGTTGAGGACAGAGAGGG			
CASP9	F	CTGGAATGCCTTCAGGAGACGGG	125	102.2	66.0
	R	GGGAGGGGCAAGACAACAGGGTG			
CASP3	F	GCTTCATTCGTCTGTGTTC	98	94.5	56.0
	R	CGAAAAAGTGATGTGAGGTA			
PCK	F	TGCTTGACTGGATGTTCAGG	178	94.5	59.3
	R	TTCCTCACCTCATCCACCTC			
FBPase	F	CTTCACCTCCTGTGTGCTTG	182	95.9	59.3
	R	CAGCTGGCTCACCATCTGTA			
G6Pase	F	GGGAGTCCAGGTGTGTGTCT	182	90.9	56.6
	R	CAGCGAAGGAGGTCAAGAAG			
FOXO1	F	CTATGAATGGCCGCTTGCTCA	164	97.3	60.0
	R	TCGTCCATATCCGTTGGTGTTG			
ACC1	F	ATCCCTCTTTGCCACTGTTG	121	102.2	57.5
	R	GAGGTGATGTTGCTCGCATA			
FASN	F	TGTGGTGCTGAACTCTCTGG	121	102.1	57.5
	R	CATGCCTAGTGGGGAGTTGT			
LPIN1	F	TCCTACGTTCCCGAGAGAAA	136	98.8	58.5
	R	TACGAGGGAACCACTTCCTG			
DGAT1	F	CACGCCTCTTCTTGGAGAAC	176	105.3	58.5
	R	AATGGTACCCACAGCCAGAC			
PPARγ	F	CCTGTGAGGGCTGTAAGGGTTT	103	100.2	59.0
	R	TTGTTGCGGGACTTCTTGTGA			
ATGL	F	CCATGATGCTCCCCTACACT	176	99.1	58.0
	R	GGCAGATACACTTCGGGAAA			
HSL	F	ATCAGAGCTGGAGCACCCTA	122	99.3	60.0
	R	GCAGAGGAGAGCAGAAAGGA			
MGL	F	AAGGTTTTTCTGGCGAAGGT	130	96.8	58.0
	R	CGTGGAAGTTCAGCTCATCA			
CPT1α	F	CATGGAAAGCCAGCCTTTAG	128	98.8	60.0
	R	GAGCACCAGACACGCTAACA			
PPARα	F	CCACCGCAATGGTCGATATG	144	104.3	59.0
	R	TGCTGTTGATGGACTGGGAAA			
SREBP1	F	AGTCTGAGCTACAGCGACAAGG	127	98.1	61.0
	R	TCATCACCAACAGGAGGTCACA			

*F, forward primer; R, reverse primer; EF1α, elongation factor 1α; TNF-α, tumor necrosis factor α; IL, interleukin; TGF-β1, transforming growth factor β1; CASP, cysteine-aspartic proteases; PCK, phosphoenolpyruvate carboxykinase; FBPase, fructose-1,6-bisphosphatase; G6Pase, glucose-6-phosphatase; FOXO1, forkhead box O1; ACC1, acetyl-CoA carboxylase 1; FASN, fatty acid synthase; DGAT1, diacylglycerol O-acyltransferase 1; PPAR, peroxisome proliferator activated receptor; ATGL, adipose triglyceride lipase; HSL, hormone-sensitive lipase; MGL, monoacylglycerol lipase; CPT1α, carnitine palmitoyltransferase 1α; SREBP1, sterol regulatory element-binding protein 1.*

The RT-qPCR temperature profile for all genes was 95°C for 3 min followed by 40 cycles of 15 s at 95°C, 30 s at Tm (Table 2) and 40 s at 72°C. After the final cycle of PCR, the melting curves were systematically monitored (65°C temperature gradient at 0.5°C/10 s from 65 to 95°C). During the detection, each sample was run in triplicate. PCR-grade water in place of the template served as the negative control. The 2^–ΔΔ*Ct*^ method was used to analyze RT-qPCR data (Livak and Schmittgen, 2001). The mRNA levels of target genes were shown as the n-fold difference relative to the calibrator.

### Histopathological, Immunofluorescence Examination, and TUNEL Assay of the Liver

All fixed liver samples were dehydrated by the standard procedures, and the samples were embedded in paraffin and cut to 6 μm sections. Liver sections were stained following the protocols of hematoxylin and eosin (H&E) staining, Sirius red staining and periodic acid Schiff staining (PAS), then observed by light microscopy (DM2500, Leica, Germany). The liver samples were identified as three phenotypes as described by Yu et al. (2018): no obvious abnormality showed regular hepatocytes morphology and clearly located cell nuclei; steatohepatitis featured as intense vacuoles in the hepatocytes resembling lipids and inflammation; hepatic fibrosis with massive collagen signal and glycogen granules.

The immunofluorescence test for activated/cleaved caspase-3 (cysteine-aspartic protease-3) was as follows (Yu et al., 2019). Liver sections were deparaffinized, rehydrated and rinsed with PBST (0.1% Tween-20 in phosphate buffered saline). Antigen retrieval was obtained by maintaining slides in citrate antigen retrieval solution (pH 6.0) in a pressure cooker for 10 min. Sections were blocked 30 min by serum-free blocking buffer (Dako, United States). Sections were incubated with the polyclonal cleaved caspase-3 antibody (Abcam, United Kingdom) overnight at 4°C. After washing with PBST, sections were incubated with Alexa Flour 555 goat anti-rabbit antibody (Life Technology, United States) for 1 h at room temperature. After 3 washes with PBST, sections were mounted with the anti-fade mounting medium that contains DAPI (Vector Laboratories, United States) for nuclei staining.

The TUNEL (TdT-mediated dUTP Nick-End Labeling) assay was carried out using a One-Step TUNEL Apoptosis Assay Kit (Beyotime, China) (Ding et al., 2018) for detecting DNA fragmentation in liver sections. The fluorescent signal was captured using a confocal microscope (LSM700, Zeiss, Germany) in merge format.

### Western Blot

The western blot analysis was carried out as described previously (Liang et al., 2019). Liver tissues were homogenized in RIPA buffer (Beyotime, China) with an added phosphatase inhibitor cocktail (Thermo Fisher Scientific^TM^, United States). The protein concentration was measured using a BCA Protein Quantification Kit (Bio-Rad, United States). Protein extracts were run on TGX Stain-Free polyacrylamide gels (Bio-Rad, United States) and blotted onto polyvinylidene fluoride (PVDF) membranes (Millipore, United States). After blocking for 1 h at room temperature, immunoblots were incubated overnight at 4°C in primary antibodies, including a loading control antibody, glyceraldehyde-3-phosphate dehydrogenase (GAPDH, Hangzhou Goodhere Biotechnology Co., Ltd., China), and target proteins including phosphorylated AKT (P-AKT, Ser473, Cell Signaling Technology, United States) and AKT (Cell Signaling Technology, United States), P-AMPKα (Thr172, Cell Signaling Technology, United States), and AMPKα (Cell Signaling Technology, United States). All the blots were then incubated for 1 h in goat anti-rabbit IgG-HRP secondary antibody (Santa Cruz Biotechnology, United States). Proteins were detected using Clarity^TM^ ECL Western Blotting Substrate (Bio-Rad, United States). Quantification was performed by ImageJ software (Rawak Software, Inc., Germany).

### Statistical Analysis

SPSS 17.0 (SPSS Inc., United States) was used to perform the statistical analysis. All data were reported as the mean value with the standard errors of the mean (mean ± SEM). The data in Tables 3–5 were firstly tested for normal distribution and homogeneity of variance and then processed one-way analysis of variance (ANOVA), followed by Duncan’s multiple comparisons. The other data were analyzed by independent *t*-test. *P* < 0.05 was considered statistically significant. The graphics were drawn by GraphPad Prism 7.0 (GraphPad Software Inc., United States).

**TABLE 3:** Effects of dietary bile acids on the survival, growth, and somatic indices of *Micropterus salmoides* (means ± SEM, *n* = 6).

	**B0**	**B80**	**B160**	**B240**	**B300**	**B600**
SR (%)	98.9 ± 0.70	97.8 ± 1.65	98.9 ± 0.70	97.2 ± 1.59	98.3 ± 1.67	100 ± 0.00
FBW (g)	34.7 ± 0.71^*a*^	35.1 ± 0.83^*a*^	34.5 ± 0.64^*a*^	37.1 ± 1.07^*a*^	39.8 ± 1.08^*b*^	36.5 ± 0.85^*a*^
WGR (%)	457 ± 12.8^*a*^	457 ± 15.4^*a*^	452 ± 11.9^*a*^	485 ± 17.6^*a*^	534 ± 14.4^*b*^	492 ± 14.0^*ab*^
SGR (% day^–1^)	2.70 ± 0.03^*a*^	2.72 ± 0.04^*a*^	2.69 ± 0.03^*a*^	2.80 ± 0.05^*ab*^	2.91 ± 0.04^*b*^	2.78 ± 0.04^*a*^
VFI (% bw day^–1^)	1.59 ± 0.02^*a*^	1.65 ± 0.02^*b*^	1.63 ± 0.02^*ab*^	1.67 ± 0.01^*b*^	1.68 ± 0.02^*b*^	1.67 ± 0.01^*b*^
FCR	0.67 ± 0.01	0.70 ± 0.02	0.69 ± 0.01	0.69 ± 0.01	0.68 ± 0.01	0.69 ± 0.01
PLV (%)	91.7 ± 4.49^*a*^	107 ± 3.80^*abc*^	113 ± 10.6^*bc*^	122 ± 7.88^*c*^	116 ± 5.89^*bc*^	96.2 ± 5.04^*ab*^
PPV (%)	46.4 ± 1.09	44.7 ± 1.17	46.4 ± 0.94	45.7 ± 0.81	47.0 ± 1.46	46.1 ± 1.00
HSI (%)	3.17 ± 0.26^*ab*^	3.53 ± 0.31^*b*^	3.47 ± 0.38^*b*^	2.58 ± 0.27^*a*^	2.57 ± 0.27^*a*^	2.41 ± 0.21^*a*^
VSI (%)	7.61 ± 0.26	8.83 ± 0.69	8.40 ± 0.50	7.72 ± 0.48	7.93 ± 0.33	7.89 ± 0.56

*SR (survival rate, %) = 100 × final fish number/initial fish number; WGR (weight gain rate, %) = 100 × (FBW−IBW)/IBW. where FBW and IBM are the final and the initial body weight of fish in the trial (batch weight divided by the number of fish); SGR (specific growth rate, % day^–1^) = 100 × [Ln (FBW/IBW)]/days; VFI (voluntary feed intake, % body weight day^–1^) = 100 × total feed consumption/[(initial total weight + final total weight)/2]/days; FCR (feed conversion rate) = FI_*abs*_/[(final total weight−initial total weight)/days]. Where, FI_*abs*_ is the daily absolute feed intake; PLV (productive lipid value, %) = lipid gain × 100/lipid intake; PPV (productive protein value, %) = protein gain × 100/protein intake; HSI (hepatosomatic index, %) = 100 × liver weight/whole body weight; VSI (viscerosomatic index, %) = 100 × visceral weight/whole body weight; Within the same row, values with different superscripts are significantly different (one-way ANOVA followed by Duncan’s test, *P* < 0.05).*

**TABLE 4:** Effects of dietary bile acids on composition of whole body and liver of *Micropterus salmoides* (wet weight %, means ± SEM, *n* = 6).

	**B0**	**B80**	**B160**	**B240**	**B300**	**B600**
**Whole body**						
Crude protein	15.7 ± 0.25	15.7 ± 0.28	16.0 ± 0.22	15.9 ± 0.15	16.0 ± 0.23	16.0 ± 0.34
Crude lipid	8.75 ± 0.35^a^	10.3 ± 0.46^abc^	10.6 ± 0.83^*bc*^	11.3 ± 0.64^*c*^	11.0 ± 0.63^bc^	9.27 ± 0.39^ab^
Moisture	69.5 ± 1.18	68.6 ± 0.78	68.4 ± 1.18	67.3 ± 0.90	68.4 ± 1.15	69.8 ± 0.41
**Liver**						
Crude lipid	2.76 ± 0.11^b^	2.47 ± 0.30^ab^	2.58 ± 0.23^ab^	2.06 ± 0.20^a^	1.98 ± 0.24^a^	2.18 ± 0.25^ab^
Moisture	62.4 ± 0.86^ab^	63.4 ± 0.86^b^	61.4 ± 0.80^ab^	60.0 ± 1.39^ab^	59.1 ± 0.86^a^	60.7 ± 1.79^ab^

*Within the same row, values with different superscripts are significantly different (one-way ANOVA followed by Duncan’s test, *P* < 0.05).*

**TABLE 5:** Effects of dietary bile acids on plasma biochemical parameters of *Micropterus salmoides* (means ± SEM, *n* = 8).

	**B0**	**B80**	**B160**	**B240**	**B300**	**B600**
**Liver functions**						
ALT (U/L)	27.9 ± 3.25^b^	28.6 ± 5.45^b^	17.6 ± 2.32^a^	15.0 ± 1.06^a^	16.5 ± 2.62^a^	18.0 ± 2.25^a^
AST (U/L)	9.68 ± 1.76^a^	11.2 ± 2.17^a^	16.5 ± 4.68^ab^	18.4 ± 6.46^ab^	22.4 ± 5.09^ab^	30.3 ± 4.96^b^
TP (g/L)	12.2 ± 1.24^a^	15.0 ± 0.73^ab^	15.5 ± 1.49^ab^	22.8 ± 3.48^bc^	26.7 ± 3.22^*c*^	24.6 ± 3.54^*c*^
ALB (g/L)	4.91 ± 0.36	5.59 ± 0.45	6.53 ± 0.59	6.39 ± 0.81	6.07 ± 0.57	5.88 ± 0.24
BUN (mmol/L)	1.30 ± 0.14	1.30 ± 0.13	1.71 ± 0.15	1.36 ± 0.07	1.67 ± 0.22	1.50 ± 0.16
**Lipid and glucose metabolism**						
GLU (mmol/L)	3.59 ± 0.92	3.64 ± 0.55	2.13 ± 0.10	3.13 ± 0.48	3.44 ± 0.57	2.49 ± 0.18
TG (mmol/L)	2.96 ± 0.68	2.51 ± 0.47	2.97 ± 0.56	3.01 ± 0.55	2.29 ± 0.46	3.69 ± 0.85
TC (mmol/L)	1.64 ± 0.22^a^	1.64 ± 0.08^a^	2.54 ± 0.47^ab^	2.67 ± 0.37^b^	1.88 ± 0.22^ab^	2.63 ± 0.31^b^
HDL-C (mmol/L)	0.22 ± 0.03^a^	0.25 ± 0.04^ab^	0.21 ± 0.04^a^	0.39 ± 0.05^*c*^	0.37 ± 0.05^bc^	0.39 ± 0.03^*c*^
HDL-C/TC	0.17 ± 0.05^ab^	0.15 ± 0.03^ab^	0.10 ± 0.02^a^	0.16 ± 0.03^ab^	0.21 ± 0.03^b^	0.16 ± 0.02^ab^

*ALT, aminotransferase; AST, aspartate aminotransferase; TP, total protein; ALB, albumin; BUN, urea nitrogen; GLU, glucose; TG, triglyceride; TC, total cholesterol; HDL-C, high-density lipoprotein cholesterol. Within the same row, values with different superscripts are significantly different (one-way ANOVA followed by Duncan’s test, *P* < 0.05).*

## Results

### Survival, Growth, and Somatic Indices

**FIGURE 1:**
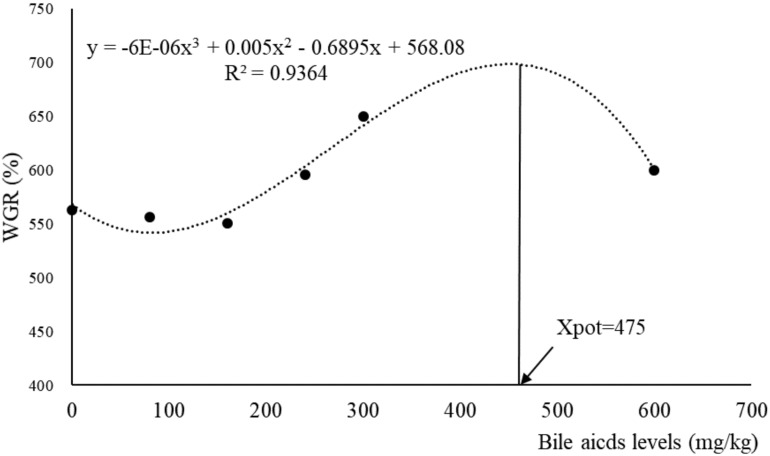
Relationship between dietary bile acids and WGR (weight gain rate) based on monistic cubic equation regression analysis.

The results of survival, growth and somatic indices were presented in Table 3. The SR of largemouth bass in all groups were above 97% and with no significant difference among groups (*P* > 0.05). The fish fed with 300 mg/kg BA had significantly higher FBW, WGR, and SGR than those in the control group (*P* < 0.05). Dietary BA improved VFI significantly (*P* < 0.05). The PLV was increased significantly in fish fed 160, 240, and 300 mg/kg BA inclusion diets when compared to the control group (*P* < 0.05). No significant effects were observed on FCR and PPV (*P* > 0.05). Based on the data of WGR, the optimal dietary BA level based on an 18.7% starch inclusion diet for largemouth bass was 475 mg/kg when estimated by monistic cubic equation regression analysis (Figure 1). With the increasing of dietary BA, HSI slightly increased from 3.17 to 3.53% then decreased to the lowest 2.41% (*P* < 0.05). The VSI was not affected by dietary BA (*P* > 0.05) (Table 3).

### Proximate Composition of Whole Body and Liver

In B240 and B300 groups, fish showed significantly higher lipid content in the whole body, but lower in liver tissue (*P* < 0.05). No significant difference in whole-body crude protein was detected among groups (*P* > 0.05) (Table 4).

### Plasma Biochemical Parameters

Plasma ALT decreased while AST increased significantly with the increase of dietary BA (*P* < 0.05). Plasma TP was improved by dietary BA and the highest levels were observed in B300 and B600 groups (*P* < 0.05). No significant differences were observed in plasma ALB, BUN, GLU, and TG (*P* > 0.05). Plasma TC enhanced significantly in B240 and B600 groups, and HDL-C and HDL-C/TC increased significantly in B300 group (*P* < 0.05) (Table 5).

### Hepatic Pathological Examination: Histology, Inflammation, and Apoptosis

The fish in B300 group had not only highest growth but also lowest HSI and hepatic lipid content, so we compared B300 with B0 groups to investigate the effect of dietary BA on histopathology and the potential regulation mechanism on glucose and lipid metabolism in the liver of largemouth bass.

**FIGURE 2:**
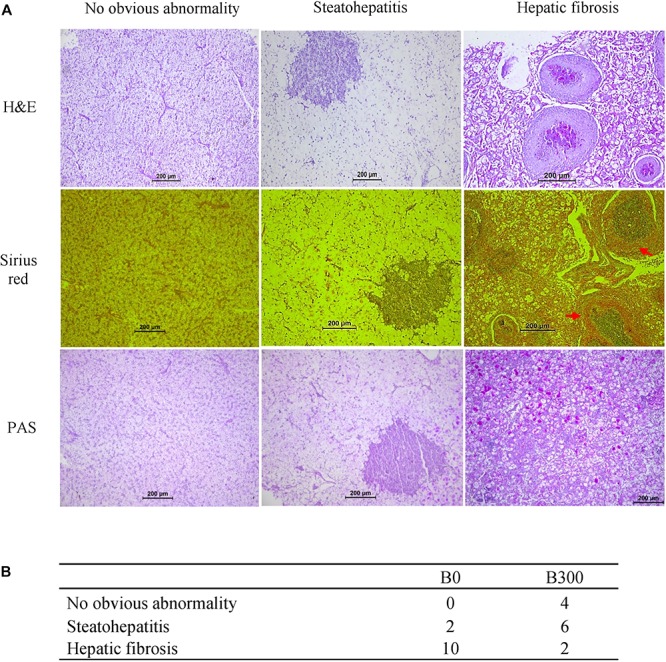
Hepatic histopathological analysis of largemouth bass in B0 and B300 groups: **(A)** Three phenotypes of hepatic histopathological examination. H&E (hematoxylin and eosin) staining for histology examination; Sirius red staining for collagen fibers (red arrow showed hepatic fibrosis); PAS (periodic acid Schiff staining) for glycogen (pink arrow showed hepatic glycogen). **(B)** Phenotypes descriptive results (*n* = 12).

Three typical phenotypes were found from hepatic samples of largemouth bass (Figure 2A). In the B0 group, two samples showed hepatic steatohepatitis, and 10 samples with serious fibrosis symptom. In the B300 group, 4 out of 12 samples were no obvious abnormality, six samples with steatohepatitis and the other two with fibrosis phenotype. The descriptive hepatic histopathological examination results of each group were shown in Figure 2B.

**FIGURE 3:**
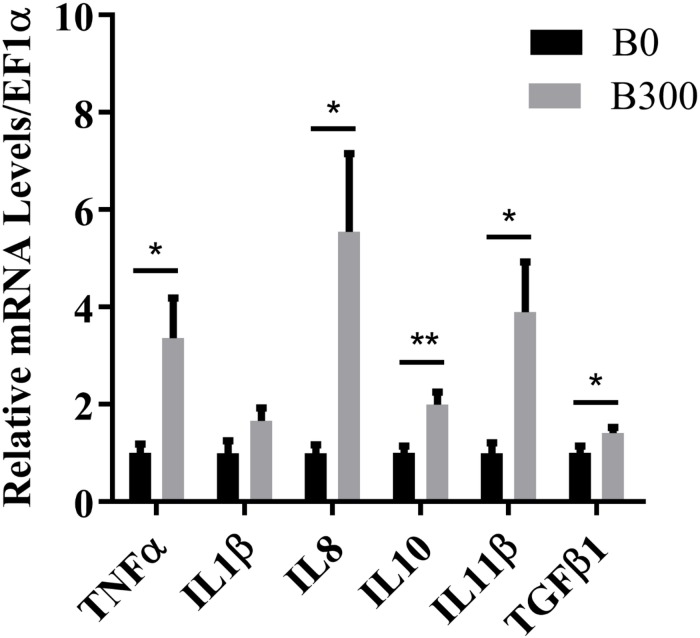
Hepatic mRNA levels of inflammatory cytokines in B0 and B300 groups. TNF-α, tumor necrosis factor α; IL, interleukin; TGF-β1, transforming growth factor β1. Values are reported as mean ± SEM (*n* = 8). Values marked with asterisks are significantly different (Independent *t*-test, ^∗^*P* < 0.05, ^∗∗^*P* < 0.01).

The mRNA levels of inflammation-related genes were presented in Figure 3. Both pro-inflammatory cytokines TNFα, IL8, and anti-inflammatory cytokines IL10, IL11β, and TGFβ1 were significantly up-regulated in B300 group (*P* < 0.05). The mRNA levels of IL1β was not affected (*P* > 0.05).

**FIGURE 4:**
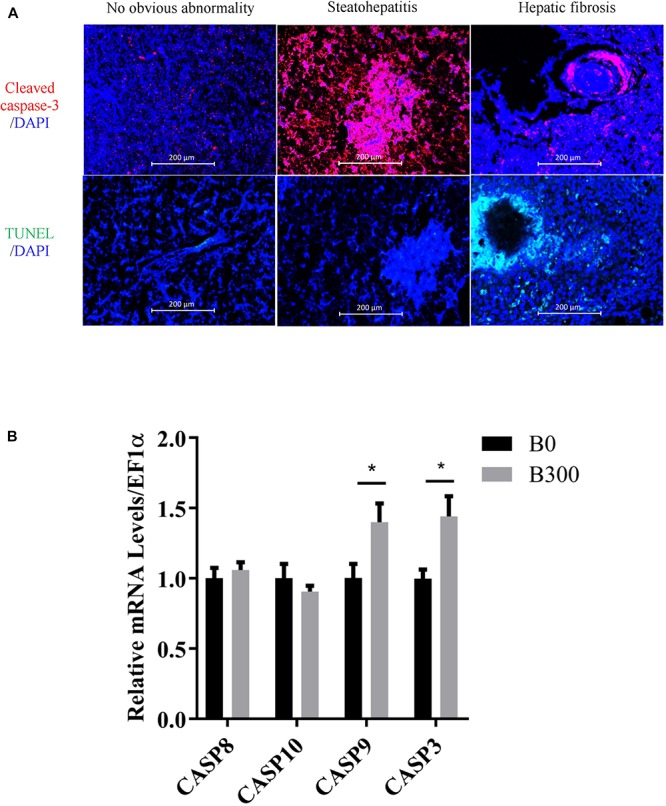
Hepatic apoptosis analysis in B0 and B300 groups: **(A)** Cleaved caspase 3 (cysteine-aspartic protease-3, apoptosis signal in red color and DAPI for nucleus) and TUNEL (TdT-mediated dUTP Nick-End Labeling) assay for cell apoptosis by immunofluorescence method. **(B)** Hepatic mRNA levels of caspases in B0 and B300 groups. CASP, cysteine-aspartic proteases. Values are reported as mean ± SEM (*n* = 8). Values marked with asterisks are significantly different (Independent *t*-test, ^∗^*P* < 0.05).

The samples with hepatic steatohepatitis and fibrosis had high expression of cleaved caspase-3 (the red fluorescence signal in Figure 4A), but only fibrosis tissues showed high-level DNA damage signals determined by TUNEL assay (the green fluorescence signal in Figure 4A). The mRNA levels of CASP9 and CASP3 increased significantly in B300 group (*P* < 0.05) (Figure 4B), which in accordance with the high expression of cleaved caspase-3 signals in this group. No significant differences were detected on CASP8 and CASP10 (*P* > 0.05).

### Glucose and Lipid Metabolism

**FIGURE 5:**
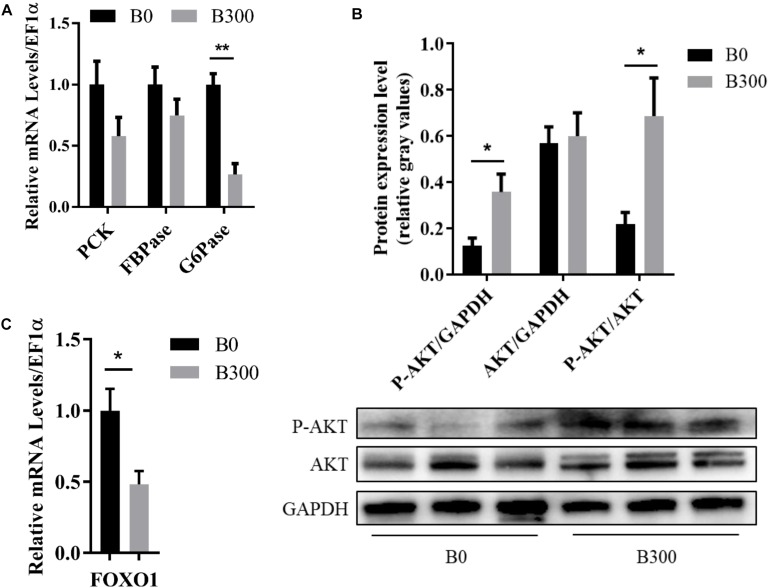
Hepatic glucose metabolism of largemouth bass in B0 and B300 groups: **(A)** Hepatic mRNA levels of gluconeogenesis-related genes in B0 and B300 groups (*n* = 8). PCK, phosphoenolpyruvate carboxykinase; FBPase, fructose-1,6-bisphosphatase; G6Pase, glucose-6-phosphatase. **(B)** Western blot of P-AKT (phosphorylated AKT serine/threonine kinase) and AKT in the liver (*n* = 3). **(C)** Hepatic mRNA levels of FOXO1 in B0 and B300 groups (*n* = 8). FOXO1, forkhead box O1. Values are reported as mean ± SEM. Values marked with asterisks are significantly different (Independent *t*-test, ^∗^*P* < 0.05, ^∗∗^*P* < 0.01).

The related glucose metabolism regulations in the liver of the fish in B0 and B300 groups were shown in Figure 5. The mRNA level of G6Pase was significantly down-regulated by BA inclusion (*P* < 0.01) (Figure 5A). Meanwhile, increased AKT protein phosphorylation (Figure 5B) and inhibited FOXO1 transcription (Figure 5C) were observed in B300 group (*P* < 0.05). There were no significant differences in the mRNA levels of PCK and FBPase between the two group (*P* > 0.05) (Figure 5A).

**FIGURE 6:**
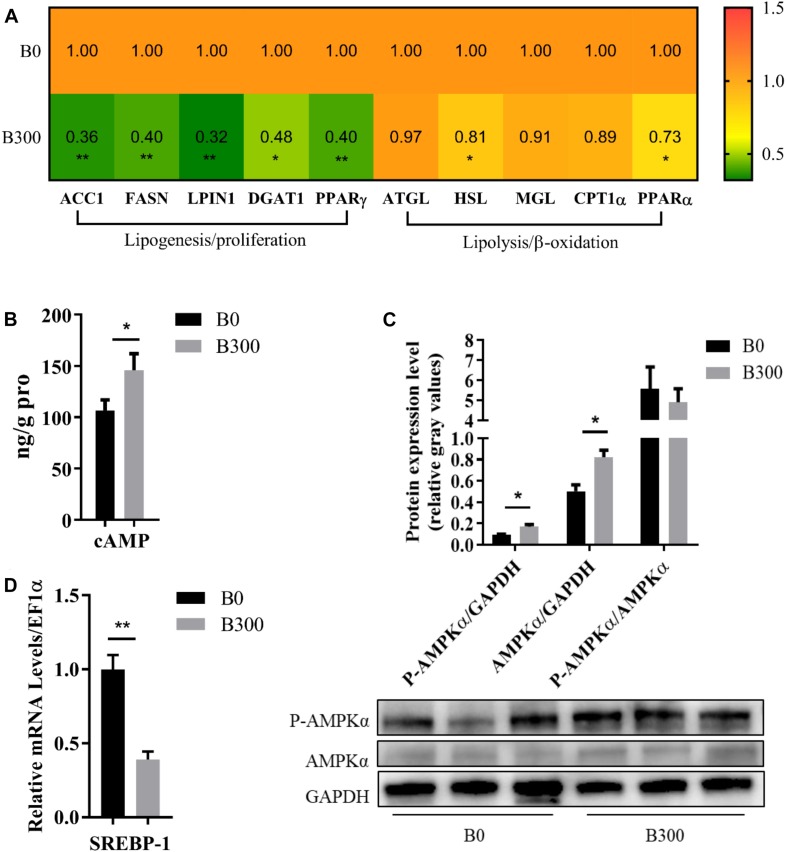
Hepatic lipid metabolism of largemouth bass in B0 and B300 groups: **(A)** Hepatic mRNA levels of lipid metabolism-related genes in B0 and B300 groups (*n* = 8). ACC1, acetyl-CoA carboxylase 1; FASN, fatty acid synthase; DGAT1, diacylglycerol O-acyltransferase 1; PPAR, peroxisome proliferator activated receptor; ATGL, adipose triglyceride lipase; HSL, hormone-sensitive lipase; MGL, monoacylglycerol lipase; CPT1α, carnitine palmitoyltransferase 1α. **(B)** Hepatic cAMP content (*n* = 8). **(C)** Western blot of P-AMPK (phosphorylated AMP-activated protein kinase) and AMPK in the liver (*n* = 3). **(D)** Hepatic mRNA levels of SREBP1 in B0 and B300 groups (*n* = 8). SREBP1, sterol regulatory element-binding protein 1. Values are reported as mean ± SEM. Values marked with asterisks are significantly different (Independent *t*-test, ^∗^*P* < 0.05, ^∗∗^*P* < 0.01).

As displayed in Figure 6A, the hepatic mRNA levels of the genes related to lipogenesis, including fatty acid synthesis genes ACC1 (*P* < 0.01), FASN (*P* < 0.01) and triglyceride synthesis genes LPIN1 (*P* < 0.01), DGAT1 (*P* < 0.05), were down-regulated significantly by more than 0.5-fold in B300 group. Dietary 300 mg/kg BA also affected the mRNA levels of lipolysis and β-oxidation-related genes. However, the heating map clearly showed that much less extent was affected, in which, the relative expression of HSL and PPARα were down-regulated by 0.19 and 0.27 compared to those in B0 group (*P* < 0.05). Significantly increased hepatic cAMP content (Figure 6B) and phosphorylation of AMPKα protein (Figure 6C) (*P* < 0.05), decreased mRNA level of SREBP1 were found in B300 group (Figure 6D).

## Discussion

Bile acids, the amphipathic end products of cholesterol metabolism, are the core member of enterohepatic circulation (Hofmann, 1999). BA as therapeutic drugs for liver diseases in mammals had been wildly investigated (Paumgartner and Beuers, 2002; Paumgartner, 2006; Fiorucci et al., 2011), but seldom studies focused on its nutrition functions. We did observe that BA supplementation enhanced the growth performance of largemouth bass fed with extruded floating diets including 18.7% starch. The growth-promotion effect of dietary BA was also indicated in turbot (*Scophthalmus maximus*) (Sun et al., 2014), rainbow trout (*Oncorhynchus mykiss*) (Yamamoto et al., 2007), and large yellow croaker (*Larimichthys crocea*) (Du et al., 2017). However, the optimal dosage varied (250–15,000 mg/kg) from fish species (Sun et al., 2014) and types of BA (Alam et al., 2001; Yamamoto et al., 2007; Du et al., 2017). In the present study, the highest WGR was obtained at 475 mg/kg BA addition in the diet of largemouth bass when estimated by a monistic cubic equation regression analysis. BA are important physiological agents for intestinal nutrients absorption, involved in the micellar solubilization of dietary lipids and act as co-factors of bile salt-dependent lipase (Hofmann, 2009; Monte et al., 2009). It was reported that dietary BA inclusion promoted feed utilization (Yamamoto et al., 2007; Jiang et al., 2018), and increased the activities of intestinal lipase (Sun et al., 2014; Jiang et al., 2018), and maltase (Yamamoto et al., 2007). Therefore, the growth-promotion effect of exogenous BA could be attributed to the increased nutrients utilization efficiency and intestinal digestive enzyme activities. Besides, BA inclusion also significantly enhanced the feed intake, which contributed to the promoted growth as well. BA functioned as chemical signals and played an important role in promoting feed ingestion (Buchinger et al., 2014) because of the high sensitivity of the olfactory neurons of fish to BA (Døving et al., 1980; Zhang and Hara, 2009). The increased feed intake by dietary BA in this study suggested the potential usage of BA as feed attractant for fish.

Carnivorous fish feed on smaller aquatic animals, where carbohydrates are rarely found in their food chain. High dietary digestible carbohydrate was recognized as the primary factor that induced MLD in largemouth bass (Goodwin et al., 2002; Amoah et al., 2008; Xu et al., 2016; Ma et al., 2019). In the present study, dietary BA increased crude lipid content of the whole body while hepatic lipid content was decreased from 2.76 (B0) to 1.98 (B300), indicating that BA acted functions on regulating the lipid metabolism and prevented lipid accumulation in the liver (Du et al., 2017). The inconsistent results of lipid content in the whole body and liver meant increased lipid storage in other tissues, like muscle or visceral adipose, the other two important lipid accumulation tissues. In the present study, no significant differences were observed on VSI. Therefore, it was supposed that dietary BA improved the filet lipid accumulation. Unfortunately, we did not take samples of filet and adipose tissues in this study. Further study needs to be conducted to explore the potential mechanism. Besides, dietary BA reduced HSI from 3.17 to around 2.5, a relatively normal value for the healthy liver of largemouth bass (Xu et al., 2016; Yu et al., 2018). Both hepatic lipid content and HSI are important indicators for the liver health of fish. Over-accumulation of lipids in the liver is associated with MLD, and high HSI is a feature for liver lesion induced by artificial diets with a high carbohydrate level in largemouth bass (Goodwin et al., 2002; Xu et al., 2016). BA supplementation potentially alleviated MLD by decreasing the hepatic lipid content and HSI of largemouth bass fed with the diets containing 18.7% starch. Besides, previous studies revealed that TP and HDL-C decreased in liver disease and HDL-C may be considered as a marker of severity of liver damage (Sharanabasappa and Nayak, 2008; Chrostek et al., 2013). The increased plasma TP and HDL-C were proofs for the beneficial effects of dietary BA on alleviating liver disease.

Metabolic liver disease encompasses a spectrum of liver abnormalities ranging from steatosis to steatohepatitis, further to fibrosis even cirrhosis (Alkhouri et al., 2009; Friedman et al., 2018). The histological examinations discovered that 10 out of 12 samples were found hepatic fibrosis in B0 group while only two fibrosis samples in B300 group, indicating that BA inclusion alleviated liver fibrosis. However, 300 mg/kg BA was not enough for curing MLD induced by 18.7% starch and 6 out of 12 samples in B300 group were still in the stage of steatohepatitis, this may be the reason why the inflammatory and apoptosis responses in B300 group kept in high levels. Inflammation are protective responses to eliminate the initial cause of cell injury and initiate tissue repair, in which inflammatory cytokines play vital roles (Karin and Clevers, 2016). Wu et al. (2018) found that grass carp showed upregulated pro-inflammatory cytokine (IL17) and anti-inflammatory cytokine (IL10) expression during the wound healing stage whereas all down-regulated in the recovery stage both in liver and gut. Wei et al. (2019) also reported that failed inflammatory response is a key reason for the severe damage in spiral valve intestine of Amur sturgeon. In the present study, the mRNA levels of both pro-inflammatory (TNFα and IL8) and anti-inflammatory cytokines (IL10, IL11β, and TGFβ1) were increased in B300 group, indicating that the fish in B300 group were undergoing hepatic self-repair. However, Inflammation are important triggers for regeneration and fibrosis (Mack, 2018). If the inflammation is not overcome or effectively treated, prolonged inflammatory responses lead to hepatic fibrosis (Lee and Friedman, 2011). Increasing the dietary BA dosage or further prolonging the feeding duration could be effective approaches to solve the MLD induced by high starch intake. The caspase family plays a very important role in mediating apoptosis, with caspase-3 being the key executive molecule (Elmore, 2007). Caspase-3 normally exists in the cytoplasm as an inactive proenzyme, which is proteolytically processed to form the active enzyme in the early stage of apoptosis (Jänicke et al., 1998). Activated caspases trigger compensatory proliferation, referred to as apoptosis-induced proliferation which maintains tissue homeostasis following massive stress-induced cell death, regenerating lost tissue (Pérez-Garijo et al., 2004; Fogarty and Bergmann, 2017). The high mRNA levels of CASP9, CASP3 in B300 group and more cleaved caspase-3 signals (apoptosis), but much less DNA damage (TUNEL) signals in most samples of B300 group (6 of 12 with steatohepatitis phenotype) implied that apoptosis induced by caspases might contribute to the damage repair in B300 group.

The positively alleviated liver fibrosis was closely related to the effects of BA on glucose and lipid metabolism in the present study. Carnivorous fish generally showed a poor ability to inhibit the gluconeogenesis pathway in response to high carbohydrate intake (Enes et al., 2011; Bou et al., 2014), Previous studies showed that the activities of PCK, FBPase, and G6Pase, the three key enzymes in gluconeogenesis, were not influenced or even increased by dietary high carbohydrate in carnivorous fish like yellow croaker (Lu et al., 2017), gilthead sea bream (*Sparus aurata*) (Enes et al., 2008a, b), and European seabass (*Dicentrarchus labrax*) (Enes et al., 2008b; Moreira et al., 2008), resulting in prolonged postprandial hyperglycemia. This may be the key reason for the poor adaptation to high dietary carbohydrate loads in carnivorous fish (Gong et al., 2015; Kamalam et al., 2017). G6Pase catalyzed the final step to complete the creation of free glucose. In this study, significantly decreased expression of hepatic G6Pase was observed in B300 group, indicating that dietary BA improved glucose homeostasis by reducing glucose production of largemouth bass. AKT is one of the well-known kinases which plays an important role in improving glucose metabolism. It was reported that BA induced AKT phosphorylation in mammals (Cao et al., 2010; Dent et al., 2010), a similar result was observed in the present study, in which AKT was activated in the liver of largemouth bass in B300 group. AKT activation is an important upstream regulatory signal and one of its downstream effectors is FOXO1 (Theofilatos et al., 2018). FOXO1 is a transcription factor, regulating several metabolic pathways in the liver, like gluconeogenesis (Yan et al., 2019). It was proved that FOXO1 promoted gluconeogenesis via activating transcription of G6Pase and PCK (Park et al., 2010; Yarushkin et al., 2013). In this study, BA inclusion activated AKT, inhibited FOXO1 and G6Pase transcription, suggesting that AKT/FOXO1/G6Pase was an important pathway via which BA inhibited gluconeogenesis. By inhibiting hepatic gluconeogenesis, dietary BA reduced the burden of glucose clearance induced by high carbohydrate intake and improved the glucose homeostasis of largemouth bass (Chiang, 2013; Dai et al., 2014).

Both the mRNA levels of ACC1, FASN (genes encoding rate-limiting enzymes in fatty acids synthesis) and LPIN1, DGAT1 (genes encoding enzymes in triglyceride synthesis) were significantly down-regulated by 0.52–0.68-fold in B300 group, proving the very significantly reduced lipogenesis in the liver. The lipolysis gene HSL and β-oxidation key transcription factor PPARα were relatively milder down-regulated by 0.19 and 0.27-fold, and the other lipolysis related genes such as ATGL, MGL, and CPT1α kept stable, indicating that BA achieved lipid-lowering effects mainly by inhibiting lipogenesis in this study. As a prototypical second messenger, cAMP is essential to mediate various cellular biological processes (Musheshe et al., 2018; Wahlang et al., 2018). BA was proved to promote (Chignard et al., 2003; Keitel et al., 2007; Brighton et al., 2015) or inhibit the synthesis of cAMP (Bouscarel et al., 1995, 1999; Kanri et al., 1996), depending on the cell types and animal species. In the present study, cAMP content was remarkably increased in the liver by dietary BA, which was consistent with the result found in hepG2 (Keitel et al., 2007) but opposite to that in hamster hepatocytes administrated BA (Bouscarel et al., 1995). The cAMP is an activator of AMPK, an important energy sensor, plays a vital role in maintaining cellular energy homeostasis (Polakof et al., 2011; Hardie et al., 2012). In mammals, enhancing AMPK phosphorylation is considered as a viable therapeutic strategy to improve metabolic disease especially non-alcoholic fatty liver disease because the activity of AMPK is reduced by inflammation, obesity and diabetes (Smith et al., 2016). There is no prior evidence about the regulation relationship of the phosphorylation status of AMPK with MLD in fish, but previous studies found that fatty acid treatment or lipid-enriched diet inhibited AMPK phosphorylation and AMPK activation suppressed lipogenesis in rainbow trout (Polakof et al., 2011; Libran-Perez et al., 2015; Velasco et al., 2018). SREBP1 is a key transcription factor regulating lipogenesis and ACC and FASN are its downstream genes (Dihingia et al., 2018). It was well documented that hepatic lipid accumulation was closely related to the induction of SREBP1 expression (Pai et al., 2013) and AMPK/SREBP pathway had critical functions in the development of MLD (Kohjima et al., 2008; Jung et al., 2011; Li et al., 2011). AMPK phosphorylation attenuated hepatic steatosis and fatty liver disease, etc., by inhibiting the expression of SREBP1, and further the inhibition of ACC and FASN (Li et al., 2011; Lee et al., 2015; Dihingia et al., 2018). The increased cAMP, up-regulated P-AMPK protein and depressed mRNA level of SREBP1, ACC, and FASN demonstrated that cAMP/AMPK/SREBP1 was an important pathway to decrease hepatic lipid accumulation by dietary BA. The results of the present study indicated that dietary BA inhibited gluconeogenesis to improve glucose homeostasis and reduced lipogenesis to prevent lipid accumulation of the largemouth bass fed with 18.7% starch inclusion diets. Activation of AKT did promote the expression of SREBP1 in some cases (Zhang et al., 2019). However, SREBP1 could be regulated by the AKT-independent pathway as well. Wang et al. (2018) found AKT was activated to inhibit gluconeogenesis while SREBP1 was suppressed in an mTOR dependent way to inhibit lipogenesis. In the present study, BA activated AKT whereas the expression of SREBP1 was inhibited by AMPK activation, supposed that AKT did not play a major role in the regulation of SREBP1.

## Conclusion

In conclusion, dietary BA enhanced the growth performance of largemouth bass significantly. The highest WGR was observed in B300 group and the optimal level of BA was estimated at 475 mg/kg by a monistic cubic equation regression analysis. BA inclusion decreased HSI and hepatic lipid content significantly. Dietary BA alleviated liver fibrosis induced by a high starch diet to steatohepatitis/recovery symptom via improving glucose and lipid metabolism which regulated by AKT/FOXO1 and cAMP/AMPK/SREBP1 pathway. The results will be not only helpful for artificial diet utilization of the species which heavily rely on chilled fish in practice, but also rewarding for MLD therapy in other animals.

## Data Availability Statement

The datasets generated for this study are available on request to the corresponding author.

## Ethics Statement

During the feeding period, the experimental fish were maintained in compliance with the Laboratory Animal Welfare Guidelines of China (Decree No. 2 of the Ministry of Science and Technology, issued in 1988).

## Author Contributions

MX and HY designed the experiments. HY, LZ, and PC carried out the experimental work. HY wrote the manuscript under the direction of MX and YQ. XL, AC, JH, XW, and YZ assisted with the experimental work.

## Conflict of Interest

The authors declare that the research was conducted in the absence of any commercial or financial relationships that could be construed as a potential conflict of interest.
